# Species mixture effects on flammability across plant phylogeny: the importance of litter particle size and the special role for non‐*Pinus* Pinaceae

**DOI:** 10.1002/ece3.2451

**Published:** 2016-10-20

**Authors:** Weiwei Zhao, William K. Cornwell, Marinda van Pomeren, Richard S. P. van Logtestijn, Johannes H. C. Cornelissen

**Affiliations:** ^1^ Systems EcologyDepartment of Ecological ScienceFaculty of Earth and Life SciencesVrije Universiteit AmsterdamAmsterdamThe Netherlands; ^2^ Ecology and Evolution Research CentreSchool of Biological, Earth and Environmental SciencesUniversity of New South WalesSydneyNSWAustralia

**Keywords:** fire, flammability, nonadditive mixture effects, phylogenetic diversity, species interactions, traits

## Abstract

Fire affects and is affected by plants. Vegetation varies in flammability, that is, its general ability to burn, at different levels of ecological organization. To scale from individual plant traits to community flammability states, understanding trait effects on species flammability variation and their interaction is important. Plant traits are the cumulative result of evolution and they show, to differing extents, phylogenetic conservatism. We asked whether phylogenetic distance between species predicts species mixture effects on litterbed flammability. We conducted controlled laboratory burns for 34 phylogenetically wide‐ranging species and 34 random two‐species mixtures from them. Generally, phylogenetic distance did not predict species mixture effects on flammability. Across the plant phylogeny, most species were flammable except those in the non‐*Pinus* Pinaceae, which shed small needles producing dense, poorly ventilated litterbeds above the packing threshold and therefore nonflammable. Consistently, either positive or negative dominance effects on flammability of certain flammable or those non‐flammable species were found in mixtures involving the non‐*Pinus* Pinaceae. We demonstrate litter particle size is key to explaining species nonadditivity in fuelbed flammability. The potential of certain species to influence fire disproportionately to their abundance might increase the positive feedback effects of plant flammability on community flammability state if flammable species are favored by fire.

## 1 Introduction

The origin of fire is tied to the origin of vascular plants, which provide two of the three essential elements for fire: oxygen and fuel (Pausas & Keeley, 2009). Since its origin, fire has accompanied terrestrial plants through their evolutionary history. Fire regime – the cumulative pattern of fires and their individual characters (fire type, frequency, intensity, season) shape organism traits, community structure, and ecosystem properties (Bond & Keeley, 2005; Bradstock, Gill, & Williams, 2012). During fire, a huge amount of stored terrestrial carbon is transferred back into the atmosphere in the form of greenhouse gases and carbon aerosols, which both influence air quality and have strong feedback effects on climate (Conard & Ivanova, 1997; Harden et al., 2000; Kasischke, Christensen, & Stocks, 1995; O'Donnell et al., 2009; Page et al., 2002). At global or continental scale, the weather can exert strong controls on fire frequency and intensity (Bradstock et al., 2012; Dale et al., 2001). As global warming proceeds, heat waves become hotter, longer, and more frequent. These shifts in climate regime are likely to increase fire risk globally (Dale et al., 2001; Field & Van Aalst, 2014; Flannigan, Krawchuk, de Groot, Wotton, & Gowman, 2009b; Flannigan, Stocks, Turetsky, & Wotton, 2009a; Kasischke et al., 1995).

Within a biome, vegetation structure and properties can greatly determine fire types and local fire regimes. One important type of fire regime globally is surface fire (Bond, 2005). The litter layer properties play an important role in determining surface fire ignition and spread (Banwell & Varner, 2014; Cornwell et al., 2015; Curt et al., 2011; Fonda, Belanger, & Burley, 1998; Papió & Trabaud, 1990; Rothermel, 1972; Schwilk & Caprio, 2011; Weber, 1991). Litter packing, which can be quantified as either packing ratio (fuel volume / litterbed volume) or as packing density (fuel mass / litterbed volume), has long been recognized as an important control on surface fire behavior (Balbi, Santoni, & Dupuy, 1999; Morvan & Dupuy, 2001; Morvan & Larini, 2001; Morvan, Méradji, & Accary, 2009; Rothermel, 1972; Viegas, 1998; Weber, 1991). Although a large body of fire behavior research has revealed the effects of bulk litter property and packing on surface fire behavior, our understanding of plant species variation in surface litter fire behavior and how plant traits difference underpin this variation is still in its infancy. Scaling from plant traits to ecosystem effects is a fundamental goal of functional ecology and fire is among the most dramatic ecosystem processes shaping vegetation and emitting CO_2_ globally.

Litterbed packing varies greatly across plant species (Cornwell et al., 2015; de Magalhaes & Schwilk, 2012; Scarff & Westoby, 2006; Stephens, Finney, & Schantz, 2004). Recently, there has been growing consensus that foliage litter particle size (the size of shed leaves, leaflets, or small branches) outweighs nonsize traits like surface area to volume ratio, litter tissue density, and chemical properties in its effect on surface fire behavior, being the first order trait determining air‐dried litterbed flammability variation across plant species through influencing litterbed packing (Cornwell et al., 2015; Schwilk, 2015). The effect of packing on flammability is mainly explained by the oxygen limitation of denser surface litter fuelbeds when surface area is not constrained. Larger litter particles create an open litterbed structure leading to greater aeration, faster flame spread rates, and higher rates of heat release (Scarff & Westoby, 2006; Schwilk, 2015).

In plant communities, species with varying leaf characteristics usually coexist. So the interactions between litters from different species are also important to scale from individual species traits to community level processes. These mixtures may not behave as additive combinations of the single species in their effects on surface litter flammability; that is, they may show nonadditive mixture effects. Only a few studies have provided evidence for nonadditive species mixture effects on flammability (van Altena, van Logtestijn, Cornwell, & Cornelissen, 2012; Blauw et al., 2015; de Magalhaes & Schwilk, 2012). According to those studies, dry litter mixtures consistently show much higher flammability than expected based on the flammability characteristics of the component species (but see Blauw et al., 2015 for occasional negative interactions in moister litterbeds); this indicates positive nonadditivity in species flammability, in which mixture flammability tends to be driven by the most flammable species when moisture is not the dominant limiting factor to fire. If only considering broad leaf and needle litters, one possible explanation for those interaction effects is that the flammable larger leaved species might increase the aeration of the mixed litterbed through structure interaction with the smaller, tightly packed litter particles mixed with them (de Magalhaes & Schwilk, 2012). In contrast, for mixtures between different litter types (e.g., leaves, twigs, moss, and lichen), the interaction effects cannot be explained by one single trait, but might be the result of multiple traits interacting, like litter tissue density, surface area to volume ratio, and fire enhancing chemical content (van Altena et al., 2012; Papió & Trabaud, 1990).

To understand the magnitude of nonadditivity, the degree of difference in terms of species traits may be important: more different species might be expected to produce larger departures from additivity in mixtures. Trait variance among species is the cumulative result of evolution. Because evolution is a conservative branching process leaving a strong legacy from the deep past, species sharing the most recent common ancestor are most likely to have similar traits composition compared to those with a distant common ancestor (Baum, 2008; Baum, Smith, & Donovan, 2005; Prinzing, Durka, Klotz, & Brandl, 2001). Being complementary with the functional approach, the degree of phylogenetic difference between species might be an alternative integrative measure for trait differences and capture some trait differences between species that are difficult to be measured for a large number of species (Cadotte, Albert, & Walker, 2013).

In this study, we use phylogenetic distance between species as a hypothesized integrative proxy for species trait differences and their effect on flammability in mixed litterbeds. We asked: “Can the strength of pair‐wise species interaction effects on litter flammability be predicted by their phylogenetic distance?” We have two hypotheses: (1) A larger phylogenetic distance is correlated with a bigger trait difference and stronger nonadditivity in flammability of the paired species. Correspondingly, a closely related species pair will behave much closer to a simple additive model of flammability. (2) Litter particle sizes, via their effects on litterbed packing, will mechanistically underpin species nonadditive mixture effects on flammability. We test these hypotheses by assessing flammability of single species and two‐species mixtures from a broad evolutionary spectrum of species in a fire laboratory. These species belong to the four large clades of land plants, but we put particular focus on litter mixtures involving gymnosperms which are often dominant in fire‐prone habitats, both today and dating back to the Mesozoic (He, Belcher, Lamont, & Lim, 2016). We sought to quantify (nonadditivity in) litterbed flammability in terms of sustainability (total burning time), combustibility (maximum temperature and integrated temperature over time), and consumability (proportion of sample burned) (Anderson, 1970; Pausas & Moreira, 2012; Schwilk, 2015).

## 2 Materials and methods

### 2.1 Species selection and litter collection

**Figure 1:**
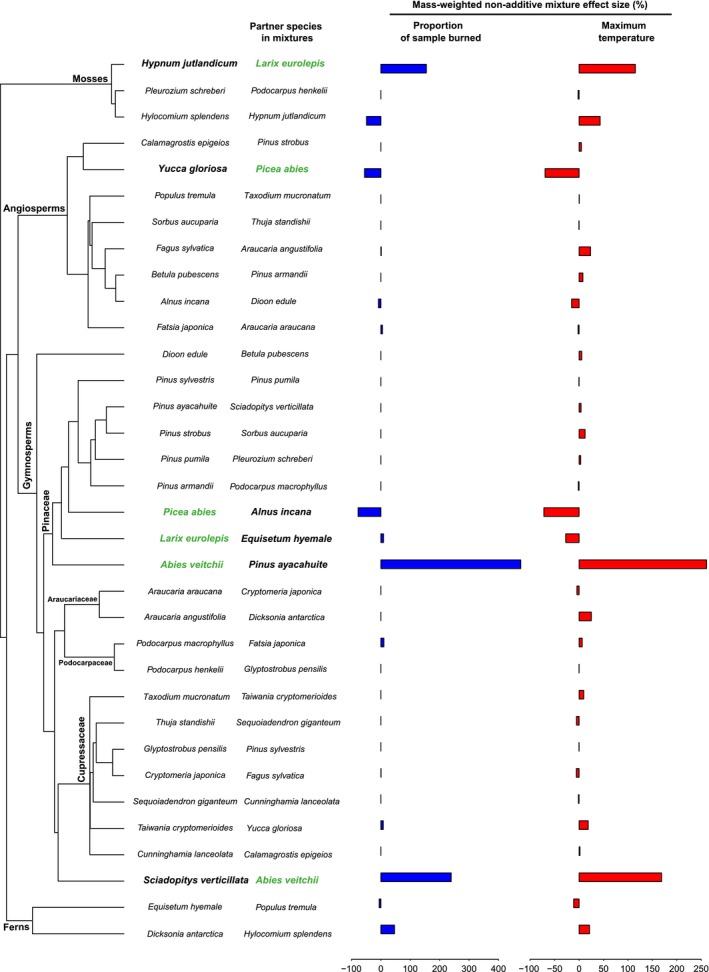
Single species phylogeny and their corresponding partner species in mixtures with mass‐weighted nonadditive mixture effect size (%) for proportion of sample burned and maximum temperature.

We selected 34 species from the four large clades of land plant: Mosses, Ferns, Gymnosperms, and Angiosperms (See Fig. 1 for species Latin names). As a follow‐up study of Cornwell et al., 2015; we also put particle focus on gymnosperms (21 species) and 17 of those species were used for both studies. For the mixtures, 34 random species pairs were selected from the pool of 34 species (Fig. 1). Most of the leaf litters were collected between 28 January and 2 March 2011 in the Netherlands: Pinetum Schovenhorst near Putten (52°15′N, 5°37′E), Pinetum Dennenhorst near Lunteren (52°05′N, 5°38′E), Pinetum Blijdestein in Hilversum (52°13′N, 5°09′E); Vrije Universiteit Amsterdam Hortus Botanicus (52°20′N, 4°51′E), Utrecht University Hortus Botanicus (52°09′N, 5°10′E), the greenhouse at Burger's Zoo (52°00′N, 5°89′E), and for bryophyte species in semi‐natural heathlands of the Netherlands Veluwe region. We also collected some species in the subarctic tundra at Abisko (68°21′N, 18°49′E), Sweden.

Litter was gathered in the form that it fell off after senescence, either as recently fallen single leaves, leaflets, or fine twigs with the leaves or needles still attached; in species that do not naturally form abscission layers, brown leaves were cut off from the living plant. After collecting, leaf litter of each species was air‐dried and then separately stored in boxes in a climate control room until use in the experimental burns.

### 2.2 Trait measurements

For all single species burns, litter particle 3D (three‐dimensional) size, litter tissue density, litter tissue moisture content, and litterbed packing ratio (total litter particle volume / litterbed volume) and packing density (total litter particle mass / litterbed volume) were measured, as these traits are likely to play important roles in determining litterbed flammability especially when fuel moisture content is low (Cornwell et al., 2015; Fonda et al., 1998; Plucinski & Anderson, 2008; Scarff & Westoby, 2006).

As the sampled plant species differ in their nature of abscission organs: needles, simple leaves, compound leaves, or small branches, the litter particle 3D size (measured as litter particle length × width × height) but not area was determined. For example, Araucariaceae and Cupressaceae species have unique litter structure that cannot be generally represented by area (See Fig. S1 for the litter particle photographs to get a visual impression of the huge variation in litter particle structures across plant species). In order to capture the average value of the particle size, the structure of the smallest, medium, and largest leaves or branch pieces were measured. We did not measure the size of Mosses as it is difficult to define what constitutes a litter particle for those plants (Fig. S1).

Litter tissue density was defined as oven‐dried mass (70°C, at least 60 hr) per litter volume. We measured litter particle volume by introducing saturated litter particles (saturated in demineralized water for 24 hr) into a beaker or graduated cylinder with a known volume of demineralized water and measuring the increase in volume. The air‐dried litter tissue moisture content was defined as the mass of water [the difference between the air dry and oven dry (70°C for at least 60 hr) leaf litter mass] per mass of air‐dried leaf litter material at equilibrium with laboratory air humidity (39% ± 4%).

For each burn, the litterbed volume was standardized using a steel mesh ring (25 cm in diameter, 3 cm in depth). Litter particles filled the ring in their natural configuration (for litters larger than the ring size, we cut them into lengths of approximately 10 cm, which we assumed to represent larger particles in that they would build fuelbeds with nonlimiting aeration). Litterbed packing density was defined as total litter air‐dried mass per ring volume. Litterbed packing ratio was defined as total litter particle volume per ring volume, where total litter particle volume was determined by total litter air‐dried mass per ring, air‐dried litter moisture content, and litter tissue density.

### 2.3 Flammability measurements

For the experimental burns, we followed the method established and described by van Altena et al. (2012) except where noted below. The standardized fire experiment was performed in the Fire Laboratory of Amsterdam for Research in Ecology (FLARE) at Vrije Universiteit Amsterdam, the Netherlands.

Two fuel types were used in the trials: mono‐specific ones (34 species) and two‐species mixtures (34 species pairs). Both fuel types were burned (when possible, with a few exceptions only) in five replications. Each replicate sample was placed in one block, which led to five blocks each of which contained one replicate of all the 68 litterbed types. Per week, one block was performed in order to minimize any effects of differences in air humidity over time. We randomized the order of the burns within each block. To record the temperature, we placed six thermocouples (1 mm thick type K thermocouple, TC Direct, Uxbridge United Kingdom): one was in the center and five were equally distributed around the ring at 6.25 cm from the center at approximately 1 cm above the surface of the fuelbed. The data recorded by the thermocouples were processed in TC Meas, a program designed in Labview (van Altena et al., 2012).

All litter samples, which were in equilibrium with the ambient air humidity in the same room, were sealed in airtight plastic bags to keep the litter humidity constant before burning. The sealing of each block was performed within one day. The mixture composition had a 50:50 distribution based on volume, meaning that a mixture contained a half filled ring litter from each of the two component species. The mass of a half ring was measured for every species in the mixture, before the two species were mixed and sealed into one airtight plastic bag. After the sample had been taken from the sealed bag and placed in the fire ring just before burning, the mixture was further homogenized. For all monoculture burns, one burn was a full ring of one species litter and samples were also weighed before burning.

Room temperature in the laboratory was controlled at 18 ± 2°C. A fume hood, which was turned on before the experiment started, held ventilation at constant and moderate speed and warmed the air from outside up to room temperature. A cotton disk drenched in 1 ml of 96% ethanol was placed in the center of the fuelbed to ignite the sample. Five different flammability parameters were measured. (1) Total burning time reflects the sustainability with which a fire burns and was measured from the temperature data as the time between ignition of the fuelbed and the complete extinction of the flame. We recorded the burning time using a stop watch during the experiment. We also calculated the burning time using the temperature data recorded during the experiment, and we defined the ignition temperature as 50°C and flame extinction temperature as 70°C. Both methods gave very similar results, and we used the results derived from the temperature data for further data analysis. (2) Fire front speed was calculated as the distance that the flame spread laterally per minute, based on the time it took for the flame to reach the ring edge or the sensors from the moment of ignition of the cotton disk. Time to the edge was measured with a stopwatch, whereas time to sensors was calculated using the temperature data recorded the thermocouples. Both measurements gave very similar results, and we report the flame speed calculated from the time to sensors here. (3) Maximum temperature was the mean of the maximum temperatures detected by the five thermocouples away from the center. (4) Temperature sum (a proxy for total heat release) (also derived from the same five thermocouples) was calculated as the sum of temperatures for each second minus a baseline temperature (defined as the average temperature before the ignition of each burning) during the whole burning period. (5) Proportion of sample burned was determined as the percent of oven‐dried mass loss during burning. The oven‐dried mass of samples before burning was calculated by the air‐dried mass of samples per ring and the air‐dried sample moisture content. The samples left after a burn were first oven‐dried at 70°C for at least 60 hr and then weighed.

### 2.4 Data analysis

We used mixture effect sizes to quantify the degree of species nonadditivity in mixtures for all flammability parameters. Mixture effect size was defined as 100 × (Observed value − Expected value)/Expected value, where observed value was the actually measured mixture flammability and expected value was the average of the flammability parameters for the two component species. Both volume‐weighted and mass‐weighted average values were calculated to test effects of weighting method on the results.

To quantify the evolutionary relatedness between the two component species in the mixture, phylogenetic distance was defined and calculated as the total branch length (Mya: million‐years) on the phylogenetic tree between two species (Cadotte et al., 2013). The phylogenetic tree was produced with the intersection of the species list from the experiment and the randomized accelerated maximum likelihood phylogenetic tree from Zanne et al. (2014).

For the comparison between phylogenetic distance and mixture effect sizes across species pairs, we use Pearson's correlation analysis. To analyze the effect of the packing ratio on the proportion of sample burned, we used a logistic regression; for maximum temperature, we used the probability density function of a normal curve (following Cornwell et al., 2015). All data analyses were processed in R (R Core Team, 2013).

## 3 Results

### 3.1 Phylogenetic distance and species mixture effects on flammability

**Figure 2:**
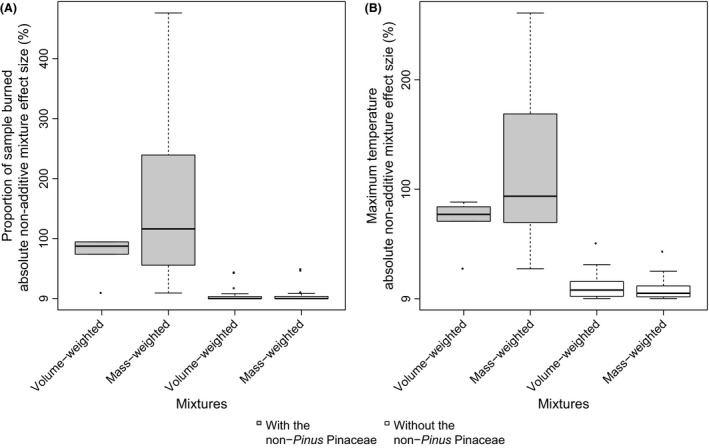
Box plot of the absolute volume‐weighted and mass‐weighted nonadditive mixture effect size values (%) for mixtures with and without the non‐*Pinus* Pinaceae of (A) percentage mass loss and (B) maximum temperature

We found that phylogenetic distance did not relate to species nonadditivity in flammability. For all flammability parameters, phylogenetic distance did not significantly correlate with mixture effect sizes, neither for volume‐weighted nor for mass‐weighted expression (Table 1). Interestingly, we found that particular mixtures involving the non‐*Pinus* Pinaceae: *Abies veitchii*,* Larix eurolepis and Picea abies* consistently show much larger or smaller effect sizes than mixtures without those species (Fig. 1, Fig. S2). Statistically, we compared the difference between mixtures with and without the non‐*Pinus* Pinaceae in absolute values of mass‐weighted and volume‐weighted mixture effect sizes (Table 2). For all flammability parameters except fire front speed, significantly higher absolute effect size values (more nonadditivity) were found for mixtures with the non‐*Pinus* Pinaceae than mixtures without the non‐*Pinus* Pinaceae (Fig. 2).

**Table 1:** Results of Pearson's correlations between phylogenetic distance and species mixture effect size (both volume‐weighted and mass‐weighted) for different flammability parameters

Volume‐weighted effect size (%)	Phylogenetic distance (Mya)			Mass‐weighted effect size (%)	Phylogenetic distance (Mya)		
	*df*	r	*p*		*df*	r	*p*
Fire front speed	32	−.13	.46	Fire front speed	32	−.02	.94
Total burning time	32	.19	.27	Total burning time	32	.04	.83
Maximum temperature	32	.13	.46	Maximum temperature	32	−.19	.28
Temperature sum	32	.14	.44	Temperature sum	32	−.16	.36
Mass loss	32	−.02	.93	Mass loss	32	−.03	.16

Significance code: *p < .05; **p < .01; ***p < .001.

**Table 2:** Statistics for two‐way ANOVA for comparing volume‐weighted versus mass‐weighted mixture effect sizes between species pairs with versus without the non‐*Pinus* Pinaceae for different flammability parameters

	Total burning time	Fire front speed	Maximum temperature	Temperature sum	Mass loss
Volume‐weighted versus Mass‐weighted					
*F*	0.52	0.10	0.76	1.24	1.42
*p*	.47	.75	.39	.27	.24
With versus without the non‐*Pinus* Pinaceae					
*F*	114.1	3.0	90.5	53.8	47.1
*p*	<.001***	.08	<.001***	<.001***	<.001***

Significance code: *p < .05; **p < .01; ***p < .001.

### 3.2 Traits, single species flammability, and species mixture effects on flammability

In our experiment, the mean litter tissue density was 0.30 g/cm^3^ with a range of 0.08–0.59 g/cm^3^_._ Mosses had the smallest tissue density. Cycad species and *Araucaria araucana* (monkey‐puzzle tree) had the densest litter tissue.

#### 3.2.1 The first order effect of litter particle size

**Figure 3:**
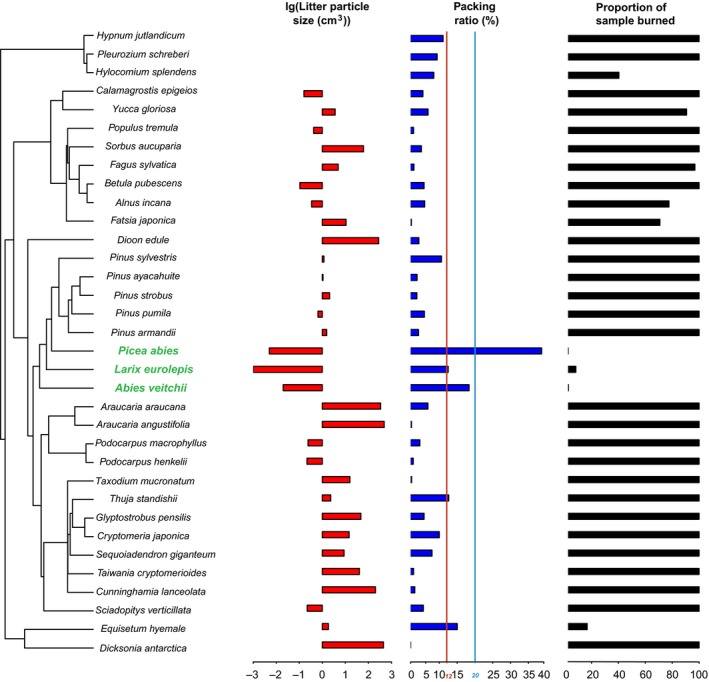
Single species phylogeny with lg [litter particle size (cm^3^)], litterbed packing ratio (%), and proportion of sample burned (%). The upper limit (12%) and lower limit (20%) of the packing ratio threshold for proportion of sample burned were indicated as red and blue vertical lines, respectively

For monoculture burns, litterbeds of most plant species burned entirely, or almost entirely. The prominent exception was the non‐*Pinus* Pinaceae species, which had <10% of samples burned. One common character of the non‐*Pinus* Pinaceae is that they singly shed small needle litters, which produce tightly packed litterbeds (Fig. 3 and see Fig. S1 for the litter particle photographs). One fern species *Equisetum hyemale* has 16.8% of sample burned, and its litterbed is tightly packed terete shape litter particles.

**Figure 4:**
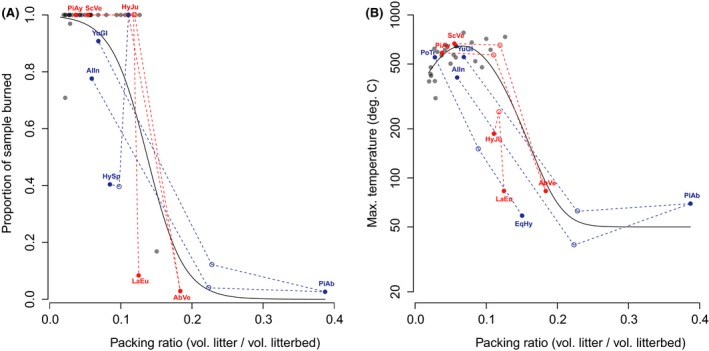
(A) Packing ratio and proportion of sample burned. Curve fit is a logistic regression for the single species burns. (B) Packing ratio and maximum temperature. Curve fit uses a normal distribution curve. In both panels, species mixtures with the three most negative and the three largest positive mass‐weighted non‐additive mixture effect sizes were indicated as red and blue open circles, respectively, and connected with their component species which is highlighted as red and blue points by dashed lines. For proportion of sample burned, species pairs with the three most negative mixture effect sizes are as follows: “*YuGl + PiAb,*” “*PiAb + AlIn,*” and “*HySp + HyJu*”; species pairs with the three largest positive mixture effect sizes are as follows: “*AbVe + PiAy,*” “*ScVe + AbVe,*” and “*HyJu + LaEu*.” For maximum temperature, species pairs with the three most negative mixture effect sizes are as follows: *“YuGl + PiAb,” “PiAb + AlIn,”* and *“EqHy + PoTr”*; species pairs with the three largest positive mixture effect sizes are as follows: *“AbVe + PoTr,” “ScVe + AbVe,” and “HyJu + LaEu.”* The meaning of the species code can be found in Table S1.

For air‐dried litter with a mean moisture content of 7% with a range from 3% to 11%, moisture content did not significantly affect litter flammability (Table 3). The relation between litterbed packing ratio and proportion of sample burned was significant in a logistic regression model (*p *< .01), with a sharp transition from flammable to nonflammable litterbeds as packing ratio increased from 12% to 20% (Fig. 4A). We defined flammable litterbeds as the ones supporting successfully fire spread to the fire ring edge and has more than 40% sample burned (Plucinski & Anderson, 2008). A similar pattern was found for maximum temperature (Fig. 4B). All non‐*Pinus* Pinaceae species had a mean litterbed packing ratio larger than 12% and were nonflammable (Fig. 3).

**Table 3:** Results of Pearson's correlation between fuelbed traits and flammability parameters for mono‐specific burns

	Packing ratio (%)			Moisture content (%)		
	*df*	r	*p*	*df*	r	*p*
Time to ignition (s)	32	−.18	.31	32	−.09	.61
Total burning time (s)	32	−.08	.64	32	−.02	.90
Flame speed (cm/min)	32	.19	.27	32	.17	.35
Maximum temperature (℃)	32	−.53	.001***	32	−.23	.18
Temperature sum (℃/min^−1^)	32	−.25	.16	32	−.13	.45
Mass loss (%)	32	−.72	<.001***	32	−.30	.09

Significance code: ***p < .001; **p < .01; *p < .05.

Almost all species pairs with extreme mixture effect sizes (the three most negative and the three most positive) included the tightly packed nonflammable *non‐Pinus* Pinaceae species. In Fig. 4, the three species pairs with the most negative effect sizes versus those with the largest positive effect sizes were highlighted and labeled. In mixtures including *Larix eurolepis* (LaEu) or *Abies veitchii* (AbVe), mixture flammability was driven by the flammable partner species, while the nonflammable *Picea abies* (PiAb) had a negative dominance effect on the mixture's flammability (Fig. 4).

#### 3.2.2 Variation among flammable species

**Figure 5:**
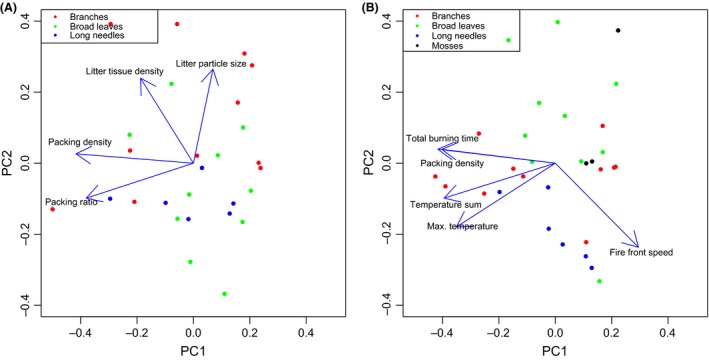
For single species burns that are flammable, (A) principal component analysis (PCA) for litter particle size, litter tissue density, packing density, and packing ratio, the first and second principal component (PC) axes explained 49.9% and 30.0% of the total variance, respectively; and (B) principal component analysis (PCA) for four flammability parameters and packing density, the first and second principal component (PC) axes explained 75.4% and 16.9% of the total variance, with each point represents a species mean, and litter particle nature: branches, broad leaves, long needles, and mosses was highlighted by different colors

**Figure 6:**
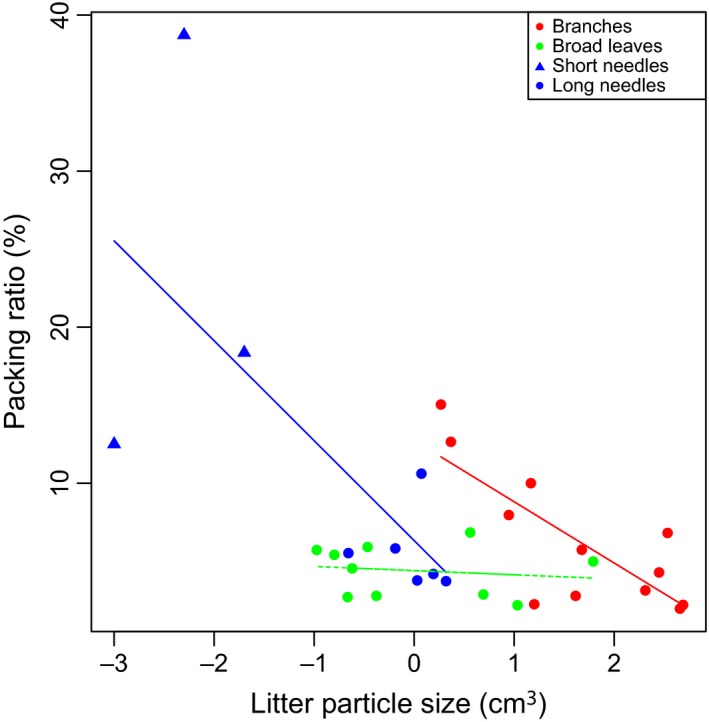
Lg [litter particle size (cm^3^)] and packing ratio (%), with litter particle of different natures: short needles (blue triangle), long needles (blue points), broad leaves (green points), and branches (red points) are indicated. Each point represents a species mean. The three short needle litter species, and the branch litter species with the biggest packing ratio value were nonflammable. Linear regression lines are fitted for needles, broad leaves, and branch litter types, with solid line indicates significant correlation (*p *< .01)

Among flammable species (i.e., excluding non‐*Pinus* Pinaceae and *Equisetum hyemale*), flammability variation had two major dimensions: sustainability (fire front speed/total burning time) and combustibility (maximum temperature/temp sum) (Fig. 5B). Along the first axis, fire either burned fast and short or slow and long. Fire sustainability was strongly correlated with litterbed packing density (Fig. 5B). Packing density increased total burning time and decreased fire front speed (Fig. 5B). Packing density was a function of litter tissue density and litterbed packing ratio (Fig. 5A). Generally, among flammable species, litterbed packing ratio was not correlated with litter particle size (Fig. 5A). However, when we categorized litter particles by their morphological structure into long needles, broad leaves, and branches, within branch litters particle size was significantly correlated with packing ratio (Fig. 6, see Fig. S1 for litter particle nature).

Both positive and negative nonadditive mixture effects in flammability were found for mixtures within flammable species (i.e., excluding mixtures including non‐*Pinus* Pinaceae and *Equisetum hyemale*), although virtually negligible in magnitude compared to mixtures involving the non‐*Pinus* Pinaceae (Fig. S2).

## 4 Discussion

We tried to use phylogenetic distance as a proxy for two‐species mixture effects on litterbed flammability. We hypothesized that larger phylogenetic distance indicated larger trait differences and greater species nonadditivity in flammability. However, in our species set, phylogenetic distance turned out to be a crude tool for estimating species nonadditivity in flammability (Table 1), and in this case, the traits and flammability relation were too particular for phylogenetic distance to be useful.

Consistent with previous laboratory and field studies (Cornwell et al., 2015; Kane, Varner, & Hiers, 2008; de Magalhaes & Schwilk, 2012; Scarff & Westoby, 2006), we demonstrate the importance of litter particle size, via affecting litterbed packing, in explaining flammability variation across 34 phylogenetically wide‐ranging species. Distinct packing ratio thresholds were found in our laboratory experiments. Near the threshold, as packing ratio increased, there was a rapid shift from flammable species to nonflammable species (Fig. 4A). Across the plant phylogeny, consistently the small needle litters from the non‐*Pinus* Pinaceae, formed litterbeds packed denser than the threshold and were thereby nonflammable. When such tightly packed small needles mixed with other, large openly packed litter particles, high nonadditivity in flammability was found (Figs 1 and 4). We suggest that litter particle size solely can explain species variation in litterbed ignitability with single small needle litter abscission as a fire suppressing trait, and underpin flammability interaction between flammable and nonflammable species. The packing threshold might vary under different environmental temperature, moisture (Blauw et al., 2015), wind conditions, and ignition patterns. For instance, previous studies find some *Abies* species are flammable (Fonda et al., 1998; de Magalhaes & Schwilk, 2012).

The threshold nature of packing effect on litterbed ignitability might explain why phylogenetic distance is not useful, as litter particle size to some extent show phylogenetic conservatism. For instance, Cupressaceae and Auracariaceae have large branch litters; *Pinus* has long needle litters, and the non‐*Pinus* Pinaceae drop solitary small needles; angiosperms has broad leaf litters; mosses have its special litter structure (Fig. S1). In our species set, phylogenetic position rather than phylogenetic distance was more informative, which was the special role for the non‐*Pinus* Pinaceae. This indicates that the nonlinear nature of the key trait effects on an ecosystem process might diminish the effect of phylogenetic diversity on ecosystem function.

The importance of litterbed packing could be explained by the aeration‐limited nature of the surface litter fire (Scarff & Westoby, 2006; Schwilk, 2015). Enough ventilation, that is, oxygen availability, is necessary for the oxidation of pyrolysates produced from thermal decomposition of the solid cellulosic fuel. When oxygen availability is limited, charring combustion becomes dominant and the relatively low temperature of charring combustion will limit further ignition of unburned litter particles and therefore inhibit fire spread (Scarff & Westoby, 2006; Weber, 1991). Above the packing threshold, litterbed ventilation is too limited to support sustainable fire spread. Below the threshold, flammability is expected to increase linearly with ventilation, saturating to a maximum value in well‐ventilated beds. Small needles build litterbeds packed denser than the threshold and thereby nonflammable, although the high surface area to volume ratio of those litterbeds might support fire spread in well‐ventilated canopy fires.

High flammability nonadditivity is most likely to be found between two species at opposite sides of the packing threshold, which is the case for mixtures involving the non‐*Pinus* Pinaceae. The negative dominance effect of *Picea abies* in mixtures presumably can be explained by their small needles filling in spaces that would otherwise be air in their paired species litterbed. This complementarity in litter particle size makes the mixed fuelbed packed denser than the threshold and become nonflammable. On the other hand, for *Larix eurolepis* and *Abies veitchii*, their small needles, tightly packed when in monospecific fuelbeds, might be spread out by the mixed large litter particles and pack less dense than the threshold, thereby becoming flammable. There are two possible explanations for these opposite interaction patterns. First, because *Picea abies* has the smallest litter length of our experimental species set, it is easy for those small and short particles to fill the space between the mixed large litter particles. Second, the structure of the large litter particles may also matter. In our experiment, coincidentally the paired species with *Picea abies*, viz. *Alnus incana* and *Yucca gloriosa*, had laminar litter structure, while partner species of *Larix eurolepis* and *Abies veitchii* either have needle‐shaped or terete litters, like *Sciadopitys verticillata, Pinus ayacahuite,* and *Equisetum hyemale*, or are mosses like *Hypnum jutlandicum* (See Fig. 1 for the species pairs and Fig. S1 for the litter particle structure). At the same packing ratio, the litterbed of long needles, terete litters, or mosses might have more small spaces than large laminar litters, and the small needles might tend to spread out rather than accumulate in those small spaces. These findings indicate that under air‐dried conditions the nature of species interaction effects on litterbed flammability can be idiosyncratic; and to better understand and predict the potential interaction patterns by litter particle traits, not only size but also litter particle three‐dimensional shape and/or surface area to volume ratio need to be considered.

### 4.1 Effects of nonsize related traits among flammable species

Among flammable species, we confirm that surface litter flammability variation has two major dimensions: sustainability (total burning time/fire front speed) and combustibility (maximum temperature/temperature sum) (Fig. 5B) (Cornwell et al., 2015; Schwilk & Caprio, 2011). A fire that burns fast facilitates fast fire spread through the landscape, with a minor soil heating effect. In contrast, a fire that has high sustainability usually produces strong damage to living tissue and soil organisms. Packing density (litter tissue density × packing ratio) explained most of the variation in fire sustainability. Among flammable litterbeds (below packing ratio threshold), when air‐dried, not only litter particle size but also litter types explained packing ratio variations (Fig. 6), which indicates the importance of surface area to volume ratio (SAV) (Papió & Trabaud, 1990). Nonadditivity in flammability among flammable species might be results of complex multiple traits interactions between the mixed species.

### 4.2 Linkage to fire in natural systems

The non‐*Pinus* Pinaceae species from genera such as *Picea*,* Larix*,* Tsuga,* and *Abies* strongly dominate the boreal forest belt and the eco‐tone between boreal and temperate forest, where these species commonly coexist with long needle coniferous and broad‐leaved tree species. The nonadditive mixture effects demonstrated here should have potentially strong influences on fire regimes and community assembly especially when burning conditions are not extreme. The positive or negative dominance effect of certain flammable or nonflammable species in mixtures involving the non‐*Pinus* Pinaceae might increase the positive feedback effects of plant flammability on local fire regimes if flammable species are also favored by fire (Bradstock et al., 2012; D'Antonio & Vitousek, 1992; He, Pausas, Belcher, Schwilk, & Lamont, 2012; Keeley, Pausas, Rundel, Bond, & Bradstock, 2011; Parsons, Balch, de Andrade, & Brando, 2015; Schwilk & Ackerly, 2001). Meanwhile, fire regime change due to fire management and global warming might strongly affect the abundance of those species in the community. We do not exclude that when canopy fire occurs small needle species might be more flammable as the high surface area to volume ratio of small needles might promote fire ignition and spread in open structured tree crown (Cornelissen et al., 2003; Wyse et al., 2016).

Flammability interaction between leaf and twig litter may be also important, both in the canopy (e.g., via crown architecture; Cornelissen et al., 2003) and in surface litterbeds. As for the latter, leaf and twig mixtures are ubiquitous feature of litterbed assembly. Leaf and twig differ greatly in SAV and lignin content and might complement in ignitability and fire sustainability. Moreover, the litter fuel we considered had not yet decomposed. Species differ significantly in litter decomposition rate; whether decomposition will diminish, exacerbate, or not affect litter packing difference among species deserves further investigation. Litter decomposition generally decrease wood density, thereby enhancing twig ignitability and fire sustainability (Zhao, Blauw, van Logtestijn, Cornwell, & Cornelissen, 2014).

Finally, both fossil records and molecular phylogenies suggest that the Pinaceae was present in the Cretaceous when surface fire regimes were important (He et al., 2016). Combine with measuring abscised particle size in the fossil record when available, the present flammability pattern of species mixtures involving the non‐*Pinus* Pinaceae reported here may also help understand paleofire behavior (See Fig. S1 for some leaf fossil photos) (Belcher, 2016; Cornwell et al., 2015; Schwilk, 2015).

In conclusion, the special leaf traits of non‐*Pinus* Pinaceae are likely to have made this lineage a major player in surface fire regimes, in past, present, and likely also future forests, with varying impact depending on the other species they grow together with.

## Funding Information

The Darwin Center for Biogeosciences, (Grant/Award Number: 142 16 3032) the Royal Netherlands Academy of Arts and Sciences (KNAW), (Grant/Award Number: CEP‐12CDP007) the Chinese Scholarship Council, the Netherlands Organization for Scientific Research (NWO), (Grant/Award Number: 047 018 003).

## Conflict of Interest

None declared.
