# Supplementary material for: Species mixture effects on flammability across plant phylogeny: the importance of litter particle size and the special role for non‐Pinus Pinaceae
Source: Ecol Evol. 2016 Oct 20;6(22):8223–34. doi: 10.1002/ece3.2451 (PMC5108272; doi:10.1002/ece3.2451)
Supplement: Supplementary file 1 [file ECE3-6-8223-s001.docx]

## Supporting Information

Article title: **Species mixture effects on flammability across plant phylogeny: the importance of litter particle size and the special role for non-*Pinus* Pinaceae**

Authors: Weiwei Zhao, William K. Cornwell, Marinda van Pomeren, Richard S. P. van Logtestijn and Johannes H.C. Cornelissen

The following Supporting Information is available for this article:

**Table S1** The list of the 34 two-species mixtures ID, code, and Latin binomial name.

| **Mixture ID** | **Mixture code** | **Mixture (Species A +Species B)** |
| --- | --- | --- |
| 1 | AbVe + PiAy | *Abies veitchii + Pinus ayacahuite* |
| 2 | AlIn + DiEd | *Alnus incana + Dioon edule* |
| 3 | ArAn + DiAn | *Araucaria angustifolia + Dicksonia antarctica* |
| 4 | ArAr + CrJa | *Araucaria araucana + Cryptomeria japonica* |
| 5 | BePu + PiAr | *Betula pubescens + Pinus armandii* |
| 6 | CaEp + PiSt | *Calamagrostis epigeios + Pinus strobus* |
| 7 | CrJa + FaSy | *Cryptomeria japonica + Fagus sylvatica* |
| 8 | CuLa + CaEp | *Cunninghamia lanceolata + Calamagrostis epigeios* |
| 9 | DiAn + HySp | *Dicksonia antarctica + Hylocomium splendens* |
| 10 | DiEd + BePu | *Dioon edule + Betula pubescens* |
| 11 | EqHy + PoTr | *Equisetum hyemale + Populus tremula* |
| 12 | FaJa + ArAr | *Fatsia japonica + Araucaria araucana* |
| 13 | FaSy + ArAn | *Fagus sylvatica + Araucaria angustifolia* |
| 14 | GlPe + PiSy | *Glyptostrobus pensilis + Pinus sylvestris* |
| 15 | HyJu + LaEu | *Hypnum jutlandicum + Larix eurolepis* |
| 16 | HySp + HyJu | *Hylocomium splendens + Hypnum jutlandicum* |
| 17 | LaEu + EqHy | *Larix eurolepis + Equisetum hyemale* |
| 18 | PiAb + AlIn | *Picea abies + Alnus incana* |
| 19 | PiAr + PoMa | *Pinus armandii + Podocarpus macrophyllus* |
| 20 | PiAy + ScVe | *Pinus ayacahuite + Sciadopitys verticillata* |
| 21 | PiPu + PlSc | *Pinus pumila + Pleurozium schreberi* |
| 22 | PiSt + SoAu | *Pinus strobus + Sorbus aucuparia* |
| 23 | PiSy + PiPu | *Pinus sylvestris + Pinus pumila* |
| 24 | PlSc + PoHe | *Pleurozium schreberi + Podocarpus henkelii* |
| 25 | PoHe + GlPe | *Podocarpus henkelii + Glyptostrobus pensilis* |
| 26 | PoMa + FaJa | *Podocarpus macrophyllus + Fatsia japonica* |
| 27 | PoTr + TaMu | *Populus tremula + Taxodium mucronatum* |
| 28 | ScVe + AbVe | *Sciadopitys verticillata + Abies veitchii* |
| 29 | SeGi + CuLa | *Sequoiadendron giganteum + Cunninghamia lanceolata* |
| 30 | SoAu + ThSt | *Sorbus aucuparia + Thuja standishii* |
| 31 | TaCr + YuGl | *Taiwania cryptomerioides + Yucca gloriosa* |
| 32 | TaMu + TaCr | *Taxodium mucronatum + Taiwania cryptomerioides* |
| 33 | ThSt + SeGi | *Thuja standishii + Sequoiadendron giganteum* |
| 34 | YuGl + PiAb | *Yucca gloriosa + Picea abies* |

**
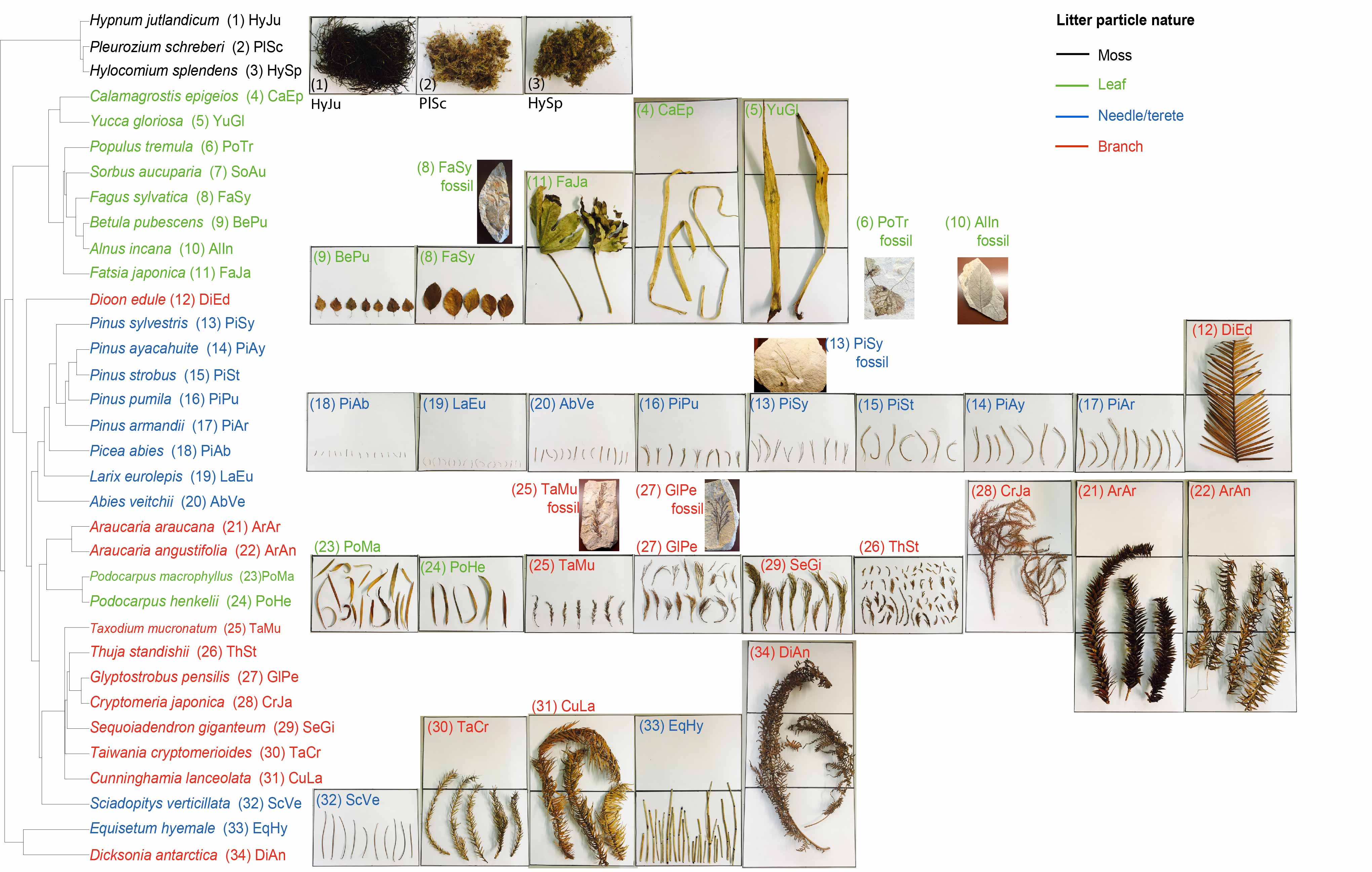
**

**Fig. S1** Single species phylogeny with litter particle photos for the 34 species used in our experiment, with the species name is indexed as species number and species code in each photo. To reflect the realistic size of the litter particle structure and make it comparable between different plant species, the background of each photo was scaled with grids (the real size of each grid equals the size of an A4 paper: 21.0 x 29.7 cm). Notice that during the fire experiments, for litter particles too big in size to fit into the fire ring (diameter 25cm, depth 3.5cm), we cut them shorter into 7-10 cm long pieces. The litter particles were categorized into four groups depending on their fresh litter structure: branches, broad leaves, mosses, and needles. We took the photos after storage of the litters for a while, so some species’ fresh litter structure has broken due to the drying of the litter and the moving of the samples. For species like *Taxodium mucronatum*, *Glyptostrobus pensilis*, *Sequoiadendron giganteum*, and *Thuja standishii*, which naturally shed branch litters, only part of their original litter structure is showed here. For *Dioon edule*, taxonomically their litter particle is a dead leaf, but here based on its structure we categorize it as branch litter type.


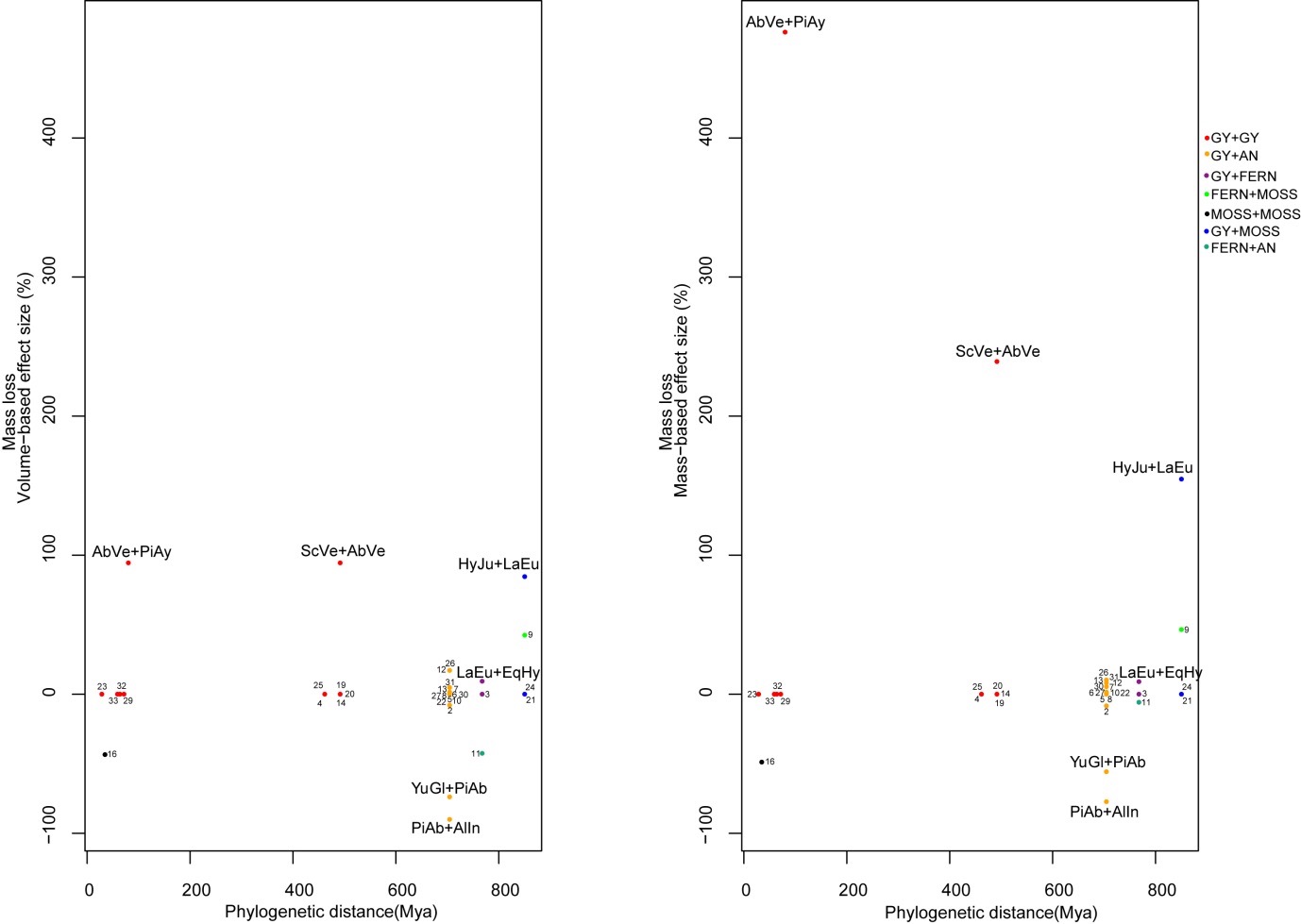


**Proportion of sample burned**

**volume weighted effect size** (%)

**Proportion of sample burned**

**mass weighted effect size** (%)

**(A)**


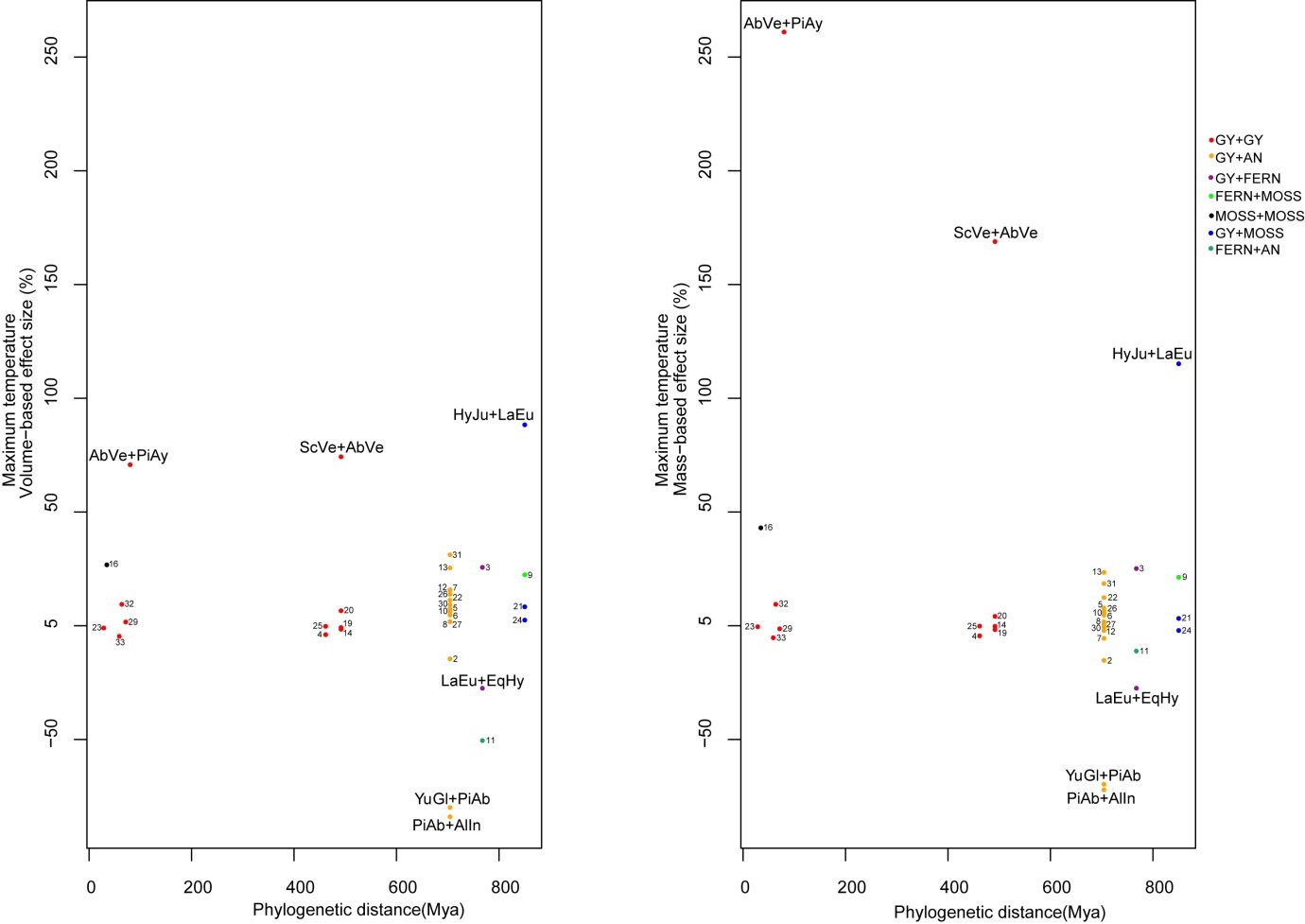


**Maximum temperature**

**mass weighted effect size** (%)

**Maximum temperature**

**volume weighted effect size** (%)

**(B)**


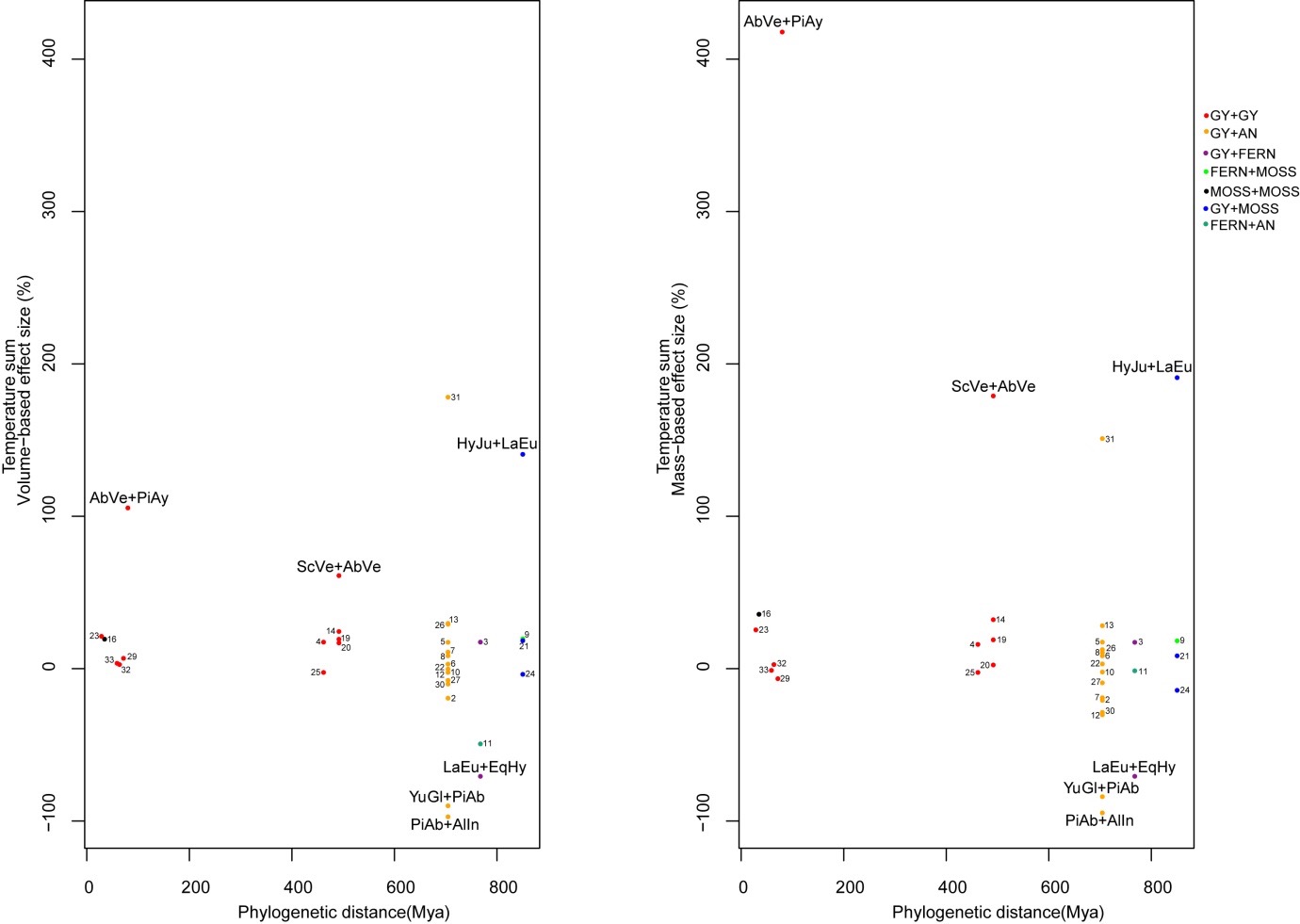


**Temperature sum**

**volume weighted effect size** (%)

**Temperature sum**

**mass weighted effect size** (%)

**(C)**


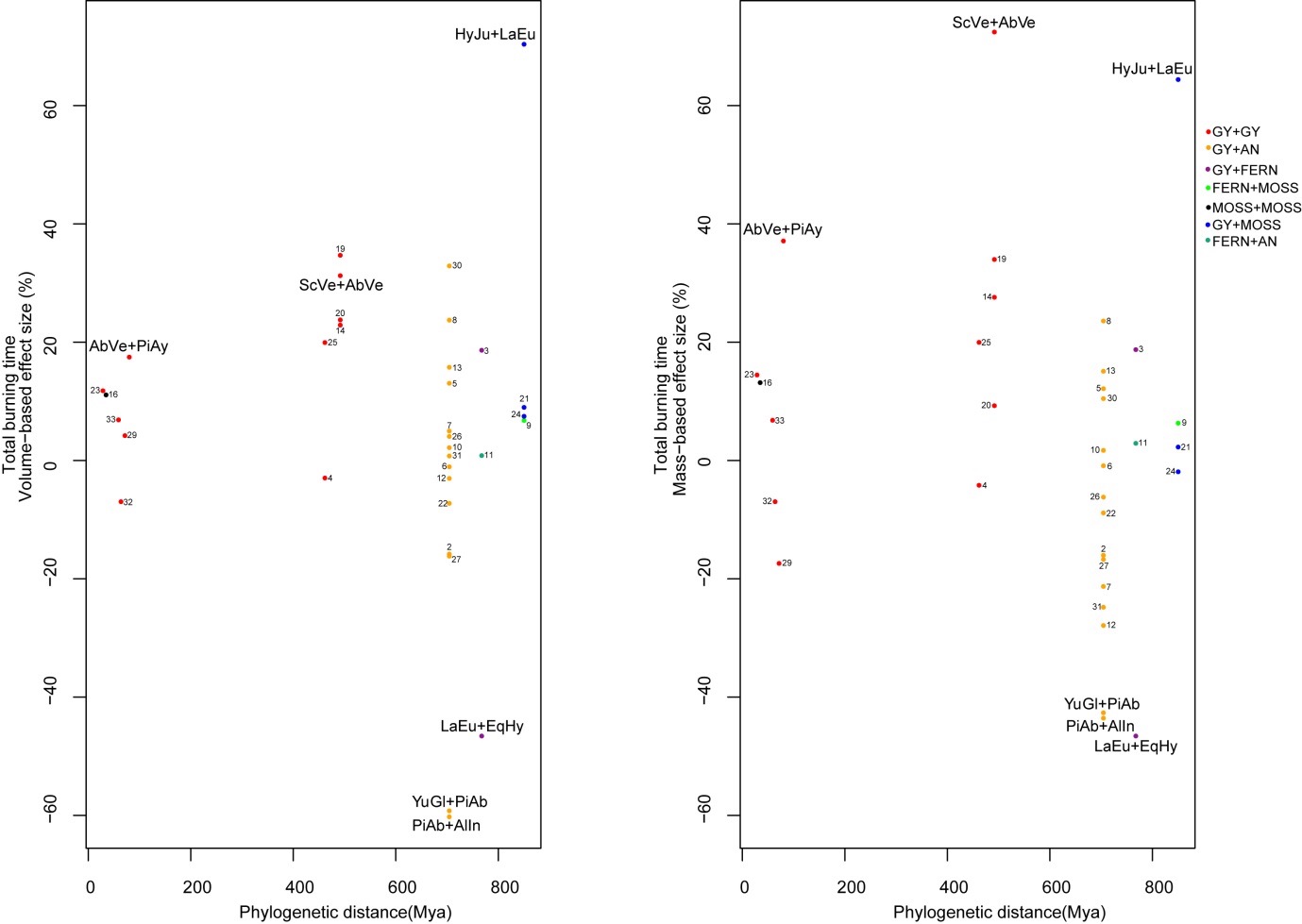


**Total burning time**

**volume weighted effect size** (%)

**Total burning time**

**mas weighted effect size** (%)

**(D)**

**Fig. S2** Scatter plots of phylogenetic distance versus species non-additive mixture effect sizes (%) (both volume-weighted and mass-weighted values) for different flammability parameters: **(A)** proportion of sample burned, **(B)** maximum temperature, **(C)** temperature sum, and **(D)** total burning time. Each point represents the mean effect size of a species pair. Depending on the division the single species belong to (Gymnosperms-GY, Angiosperms-AN, Ferns-FERN, or Mosses-MOSS), all the species pairs were categorized into seven groups: GY+ GY, GY+AN, GY+FERN, FERN+MOSS, MOSS+MOSS, GY+MOSS, and FERN+AN, which was specified by different colors. Mixtures including species of the non-*Pinus* Pinaceae clade are denoted by the component species code: “AbVe + PiAy” ( “*Abies veitchii* + *Pinus ayacahuite*”), “ScVe + AbVe” (“*Sciadopitys verticillata* + *Abies veitchii*”), “HyJu + LaEu” (“*Hypnum jutlandicum* + *Larix eurolepis*”), “LaEu + EqHy” (“*Larix eurolepis* + *Equisetum hyemale*”), “YuGl + PiAb” (“*Yucca gloriosa* + *Picea abies*”), and “PiAb + AlIn” (“*Picea abies* + *Alnus incana*”). For other mixtures, only the species mixture ID are shown. The meanings of those species mixture ID can be found in the Table S1.
